# The potential of effector-target genes in breeding for plant innate immunity

**DOI:** 10.1111/1751-7915.12023

**Published:** 2012-12-27

**Authors:** Fleur Gawehns, Ben J C Cornelissen, Frank L W Takken

**Affiliations:** 1 Department of Molecular Plant Pathology, Swammerdam Institute for Life Sciences, University of AmsterdamScience Park 904, 1098 XH, Amsterdam, the Netherlands; 2 Centre for Biosystems GenomicsDroevendaalsesteeg 1, 6708 PD, Wageningen, the Netherlands

## Abstract

Increasing numbers of infectious crop diseases that are caused by fungi and oomycetes urge the need to develop alternative strategies for resistance breeding. As an alternative for the use of resistance (*R*) genes, the application of mutant susceptibility (*S*) genes has been proposed as a potentially more durable type of resistance. Identification of *S* genes is hampered by their recessive nature. Here we explore the use of pathogen-derived effectors as molecular probes to identify *S* genes. Effectors manipulate specific host processes thereby contributing to disease. Effector targets might therefore represent *S* genes. Indeed, the *Pseudomonas syringae* effector HopZ2 was found to target MLO2, an *Arabidopsis thaliana* homologue of the barley *S* gene *Mlo*. Unfortunately, most effector targets identified so far are not applicable as *S* genes due to detrimental effects they have on other traits. However, some effector targets such as *Mlo* are successfully used, and with the increase in numbers of effector targets being identified, the numbers of *S* genes that can be used in resistance breeding will rise as well.

## Recessive traits can be used in breeding for resistance against pathogenic fungi and oomycetes

Globalization is one of the most important modern comforts of human society. However, it also negatively affects our daily life in ways that, at first sight, are not clearly connected with it. Over the last decades a steady increase in virulence of plant-infecting fungi and oomycetes has been observed. This increased pathogen virulence causes dramatic losses in the yields of crops, such as rice or wheat, locally resulting in a complete loss of harvest (Fisher *et al*., 2012). This development, which seriously threatens our crops, relates to an increase of genetic exchange between formerly geographically separated fungi and oomycetes. The accelerated macro-evolutionary genesis of new genotypes is, among other factors, caused by worldwide trading and international travel (Fisher *et al*., 2012). Even if biosecurity would be enhanced worldwide to slow down the rapid increase in pathogenicity, it would still not abolish the persistent need for new control measures for plant diseases.

Fungal and oomycete plant diseases are generally controlled with the help of fungicides, soil management and resistance breeding (McDonald and McDermott, 1993; Lazarovits, 2001). Traditional resistance breeding is based on the introgression of dominant resistance genes (*R* genes) from wild species into elite varieties. Since *R* gene-mediated resistance is based on recognition of a single elicitor, the frequency of resistance breakdown is typically high. Therefore a continuous influx of novel resistance genes in breeding programmes is required. To break this boom-and-bust cycle, susceptibility genes (*S* genes) have been proposed as an alternative to *R* genes in resistance breeding (Gust *et al*., 2010). *S* genes encode plant proteins that are manipulated by pathogens in order to facilitate their proliferation thereby promoting disease development. Hence, removal or inactivation of an *S* gene will impair the pathogens’ ability to cause disease. This type of plant immunity has the potential to be more durable (Gust *et al*., 2010). Indeed, the *mlo* locus of barley, one of the best-described recessive resistances, has been introgressed already in the 1940s (Jorgensen, 1992). This gene still confers durable broad-spectrum resistance against powdery mildews since its widespread use in the 1980s (Ortiz *et al*., 2002). *Mlo* encodes a plasma-membrane protein involved in vesicle-associated processes, which is essential for the powdery mildew to cause infection (Collins *et al*., 2003; Schulze-Lefert, 2004; Panstruga, 2005). Notably, *Mlo* is conserved throughout several plant species and recessive *mlo-*mediated powdery mildew resistance has also been identified in tomato and *Arabidopsis thaliana* (Consonni *et al*., 2006; Bai *et al*., 2008). Other examples of recessive resistance genes are *eIF4E* and *eIF4G*, conferring primarily resistance to potyviruses (Duprat *et al*., 2002; Ruffel *et al*., 2002) and *xa5* and *xa13,* which are active against *Xanthomonas oryzae* in rice (Ogawa *et al*., 1987; Zhang *et al*., 1996; Iyer and McCouch, 2004).

## Effectors from pathogens can be used as guides to identify *S* genes

Most *S* genes currently used in agriculture have been identified in screens for recessive resistances in wild germplasms (Bai *et al*., 2005). As an alternative approach, mutagenized M2 populations have been screened for loss-of-susceptibility mutants towards the pathogen of interest. These screens are often done in genetically well-defined model species, such as *A. thaliana –* a brassicales representative – due to the relative ease of identifying and cloning of the affected gene. Such screens yielded, for instance, six *DOWNY MILDEW RESISTANT* (*DMR*) (Van Damme *et al*., 2005) and six *POWDERY MILDEW RESISTANT* (*PMR*) (Vogel and Somerville, 2000; Vogel *et al*., 2004) loci. A subset of these genes has been mapped and functionally analysed for their ability to function as negative regulators of the plant defence (Vogel *et al*., 2002, 2004; Consonni *et al*., 2006; van Damme *et al*., 2008). A functional screen has the advantage that mutations causing strong pleiotropic effects, or even lethality, will automatically be discarded. A drawback is that multi-copy *S* genes, or genes with a redundant function, are likely to be missed. Over the last 15 years about 30 *S* genes have been identified; unfortunately, only few of these genes have the potential to be used in commercial breeding programmes because they also affect other traits, such as yield or plant vigour (Pavan *et al*., 2010).

Because of the relative low success rate of genetic strategies to identify suitable *S* genes an alternative method is explored here. Since many *S* genes encode proteins manipulated by a pathogen, the pathogen might be used as a guide to identify S proteins. Pathogens manipulate their host via effector proteins that interfere with host processes. Therefore, identification of plant effector targets that are insensitive towards the activity of the effector could provide insensitivity towards the pathogen (Hogenhout *et al*., 2009). Hence, *S* genes and effector targets might represent the same genes (Pavan *et al*., 2010). Indeed, the virulence function of the *Pseudomonas syringae* effector HopZ2 was found to require *A. thaliana* MLO2 (Lewis *et al*., 2012), a functional orthologue of barley Mlo (Consonni *et al*., 2006). Interestingly, independent *mlo2* knockouts were found to vary in their resistance levels to *P. syringae*. Whereas two independent T-DNA insertion lines became resistant (Lewis *et al*., 2012), a third insertion mutant and a point mutation mutant were not affected in their susceptibility towards this pathogen (Vogel and Somerville, 2000; Consonni *et al*., 2006). However, the latter mutants were resistant towards the powdery mildew *Golonivomyces orontii*. This difference shows that the type of mutation in the *S* gene can determine the outcome in resistance. This effect could also be used as an advantage with regard to pleiotropic effects as discussed later.

Besides identification of known *S* genes, also new candidates have been identified in effector-target screens. An example is the *Xanthomonas campestris* AvrBs3 effector that targets the promotor of the *upa20* gene from pepper, thereby promoting disease development by altering the expression of a transcription factor (Kay *et al*., 2007; Zhou and Chai, 2008). This atypical example, where the effector target is not a protein but a promotor region, shows the versatility of *S* genes. Both the HopZ and AvrBs3 example show that effectors can be used as guides to identify known and novel *S* genes conferring disease resistance.

## Identification of fungal and oomycete effector targets requires a tailor-made approach

Far less effector targets have been identified for plant pathogenic fungi and oomycetes than for bacteria. The reason for this is because the former generally have more complex lifestyles, larger genomes and lower accessibility to genetic approaches, such as transformation and targeted gene knockout. Functional genomics enabled the rapid identification of up to hundreds of effector candidates from fungi and oomycetes (Hogenhout *et al*., 2009). Depending on the pathogen analysed most of those proteins suppress plant immunity, indicating a redundant function. However, suppression has mostly been studied in artificial and heterologous systems and the *in vivo* function of most effectors still has to be determined with gene knockouts in the pathogens (Bozkurt *et al*., 2012). Effectors that have a clear virulence function are prime candidates to identify potential *S* genes. Such key effectors can be selected with the help of ‘effector detector’ screens, comparative genomics or *in vivo* studies (Alfano, 2009; see examples below). Most effector targets have been discovered using protein–protein interaction assays, but targets have also been predicted based on effector structure, their *in vivo* expression pattern or localization, or their biochemical activities (Alfano, 2009). Ideally, several of these effector characteristics are unveiled before the interaction study of choice is commenced. Also information about the pathogen lifestyle and the host immune system may play crucial roles in identifying the genuine target from a list of candidates. Hence, there is no universal strategy to identify effector targets. The strategy chosen to identify effector targets depends on the identity and function of the effectors and the plant-pathosystem being studied.

## Functional insights in fungal and oomycete effector targets identify *S* genes

So far, only a handful of fungal and oomycete effector targets have been identified (Table 1). They include both plant- and pathogen-derived molecules. For example, the *Cladosporium fulvum* effectors Avr4 and Ecp6 bind fungus-derived chitin oligomers thereby protecting it from plant chitinases and preventing chitin-triggered immunity (van den Burg *et al*., 2004, van den Burg *et al*., 2006, de Jonge *et al*., 2010). Among the effector plant targets there are several proteins with a positive regulatory function on the plant immune system, and these can therefore not be used as *S* genes. One such example is the haem-dependent peroxidase POX12 from corn that is targeted by the apoplastic effector Pep1 from *Ustilago maydis* (Hemetsberger *et al*., 2012). Transcriptomics and microscopical studies of the Pep1 knockout indicated a role of the effector in the apoplast (Doehlemann *et al*., 2009). Later Hemetsberger and colleagues (2012) showed via a biochemical approach that Pep1 is inhibiting POX12. Haem-dependent peroxidases are typically involved in the production of reactive oxygen species (ROS), an essential component of the early plant defence response, and their suppression enables the fungus to establish a biotrophic interaction with the host.

**Table 1:** Selection of plant effector targets and their method of identification

Effector	Pathogen	Host	Virulence target	Identified by	Reference
Avr4	*Cladosporium fulvum*	Tomato	Chitin	Structure prediction and binding studies	van den Burg *et al*. (2004)
Ecp6	*C. fulvum*	Tomato	Chitin	Structure prediction and binding studies	de Jonge *et al*. (2010)
Pep1	*Ustilago maydis*	Corn	POX12	Transcriptomics, fluorescence complementation	Hemetsberger *et al*. (2012)
Avr2	*C. fulvum*	Tomato	Rcr3	Genetics 0026; co-IP (*N. benthamiana*, *Pichia pastoris*)	Dixon *et al*. (2000); Rooney *et al*. (2005)
Avrblb2	*Phytophthora infestans*	Potato	C14	Co-IP (*N. benthamiana*)	Bozkurt *et al*. (2011)
PopP2	*Ralstonia solanacearum*	Wide range	RD19	FLIM	Bernoux *et al*. (2008)
EPIC1/EPIC2	*P. infestans*	Potato	Rcr3	Co-IP (*N. benthamiana*, *Escherichia coli*)	Song *et al*. (2009)
Gr-VAP1	*Globodera rostochiensis*	Tomato	Rcr3	Yeast two-hybrid	Lozano-Torres *et al*. (2012)
Avr3a	*P. infestans*	Potato	CMPG1	Yeast two-hybrid	Bos *et al*. (2010)

Co-IP, co-immunoprecipitation; FLIM, fluorescence lifetime imaging,

Various apoplastic effectors of different oomycetes and fungi target different papain-like cysteine proteases (PLCPs) (Kaschani *et al*., 2010). Some of these effectors, like Avr2 from *C. fulvum,* inhibit protease activity of specific PLCPs (Kruger *et al*., 2002; Rooney *et al*., 2005). Others prevent their secretion to the apoplast by retaining the PLCP in the cytoplasm, as has been shown for the *Phytophthora infestans* effector Avrblb2 (Bozkurt *et al*., 2011) and the *Ralstonia solanacearum* effector PopP2 (Bernoux *et al*., 2008). The Avrblb2 plant target was identified by *in planta* co-immunoprecipitation (Co-IP) from *Nicotiana benthamiana* leaves followed by mass spectrometric analysis. Five specific interacting plant proteins were found and among them was the C14 protease belonging to the class of PLCPs. The functional role of Avrblb2 was unravelled by microscopy studies showing inhibition of C14 secretion (Bozkurt *et al*., 2011).

In the apoplast of tomato the *C. fulvum* effector Avr2 targets Rcr3, which is also an PLCP (Kruger *et al*., 2002; Rooney *et al*., 2005). Rooney and colleagues (2005) confirmed their interaction using activity-based protease profiling and co-immunoprecipitation assays. Interestingly, Rcr3 also serves as a target for effectors from other pathogens. For example, both the *P. infestans* cystatin-like effectors EPIC1 and EPIC2B (Song *et al*., 2009) and the *Globodera rostochiensis* effector Gr-VAP1 (Lozano-Torres *et al*., 2012) exert their virulence function via Rcr3. These examples show that at least some PLCPs have the potential to be used as *S* gene.

Another interesting effector target was identified upon screening Y2H cDNA libraries made from *P. infestans* infected potato plants with the Avr3a effector. This effector specifically suppresses *P. infestans* INF1 triggered cell death (ICD) (Bos *et al*., 2006). Avr3a interacts in yeast and *in planta* with CMPG1, an E3 Ubiquitin (Ub) ligase (Bos *et al*., 2010). The ubiquitin proteasome pathway fulfils crucial functions in plant defence and several E3 Ub ligases have been found to play both positive and negative roles in immunity (Devoto *et al*., 2003). CMPG1 is required for defence response triggered by several *R* genes, the basal immune system and for ICD (Gilroy *et al*., 2011). Although CMPG1 has a positive regulatory function in tobacco and tomato, a mutation of the rice CMPG1 homologue *Spl11* confers resistance towards *Magnaporthe grisae* and *X. oryzae* revealing its potential use as an *S* gene (Yin *et al*., 2003). In line with this observation, a reduction in sporulation of *P. infestans* was observed in *CMPG1* silenced *N. benthamiana* plants (Bos *et al*., 2010). It will be interesting to test whether a *CMPG1* knockout in potato exhibits increased resistance against *P. infestans.* Besides *CPMG1* also the *A. thaliana* E3 Ub ligases Pub21, 22 and 23 were found to be negative regulators of immunity, and *pub21/pub22/pub23* mutants show spontaneous cell death and increased resistance against biotrophic pathogens (Trujillo *et al*., 2008). These findings make E3 Ub ligases interesting candidates to act as potential *S* genes.

## Pleiotropic effects limit the application of *S* genes in resistance breeding

When an effector target represents a potential *S* gene, its applicability for breeding has to be determined. Pleiotropic effects, such as dwarfism or spontaneous necrotic lesions, are a common problem for the application of *S* genes in breeding (Pavan *et al*., 2010). Furthermore, a gene that confers susceptibility to one pathogen might mediate resistance to another (Jarosch *et al*., 1999).

As discussed above, in a specific context *CMPG1* might be considered an *S* gene. However, since *CMPG1* is also necessary for basal immunity (Gilroy *et al*., 2011), application of *cmpg1* in potato resistance breeding does not seem obvious, because the plants are likely to become (hyper)sensitive to other pathogens. This trade-off between increased resistance to one, but increased susceptibility to another pathogen is one of the major drawbacks for the application of *S* genes in recessive breeding. It also applies for barley *mlo* resistance: while *mlo* confers resistance against biotrophic powdery mildew it enhances susceptibility towards necrotrophic fungi such as the rice blast fungus *M. grisae* and *Bipolaris sorokiana* (Jarosch *et al*., 1999; Kumar *et al*., 2001). Therefore, the use of *mlo* is not recommended in areas where rice and barley are grown in close proximity (Jarosch *et al*., 1999).

Also among *P. syringae* effector targets, the majority of *S* genes cannot be used because of their pleiotropic effects. MPK4 is a negative regulator of the salicylic acid (SA) response, which is required for resistance to many biotrophic pathogens. Therefore, loss of MPK4 function in *A. thaliana* leads to increased resistance against *P. syringae* and the oomycete *Peronospora parasitica* due to an accumulation of SA, but also to dwarfism and spontaneous lesions (Petersen *et al*., 2000). Similar symptoms have been reported in soybean after silencing of one of the two *MPK4* homologues (Zhang *et al*., 2000). Surprisingly, silencing of *MPK4* in tomato did not lead to a phenotype (Chen *et al*., 2009). Nevertheless, MPK4 function also affects the response to ethylene, hence leading to increased susceptibility towards the necrotrophic fungus *Alternaria brassicola* (Brodersen *et al*., 2006). This result makes the application of *mpk4* in recessive resistance breeding unlikely.

Another example concerns RIN4, an effector target and important component of basal immunity in *A. thaliana.* A *rin4* mutant exhibits increased resistance to *P. parasitica* and *P. syringae*, indicating a function for this gene as a negative regulator of basal resistance (Mackey *et al*., 2002). Silencing *rin4* in tomato enhanced resistance against *P. syringae* carrying *avrPto*, but surprisingly growth of *P. syringae* lacking *avrPto* was unaffected (Luo *et al*., 2009). Hence*,* the ability of *rin4* to mediate *P. syringae* resistance is race-specific restricting its potential use as *S* gene.

Some effector targets are monitored by R proteins, which complicate their use in breeding. Besides a negative regulatory role in basal immunity, *Rin4* has a positive regulatory function in the presence of the *R* gene *Rps2.* A knock-down of Rin4 triggers activation of the *RPM1* gene in *A. thaliana* (Mackey *et al*., 2002). Likewise, a *Rcr3* mutant shows an autoimmune phenotype due to erroneous activation of the Cf-2 protein (Kruger *et al*., 2002). Next to the pleiotropic effects described above, some *S* genes negatively affect abiotic stress tolerance. For example, silencing of *OsMAPK5* leads to enhanced resistance to *M. grisae* and *Burkholderia glumae,* but at the same time reduces plant tolerance to cold, drought and salt (Xiong and Yang, 2003).

In summary, on a case-to-case basis, taking into account the genetic background of the plant and pleiotropic effects resulting from the mutation in the *S* gene, it has to be analysed whether an effector target has the potential to be used as an *S* gene in plant breeding.

## Conclusions

Compared with *R* genes, *S* genes provide a potentially more durable type of plant immunity. The retrieval of Mlo as an effector target shows the potential use of effector proteins in the identification of *S* genes. Also other effector targets, such as RIN4, MPK4 and CMPG1, fit the criteria of being an S protein as their knockouts confer enhanced resistance to specific bacteria, fungi and oomycetes. However, their direct application in crops is hampered by the pleiotropic effects often observed in the knockouts. Possibly, specific mutations in an effector target could reduce the pleiotropic effects while maintaining its ability to confer resistance as exemplified by *mlo2.* Identification of more effector targets will increase the number of *S* genes that can be used in resistance breeding.

## Conflict of interest

None declared.

## Funding Information

The Takken lab is supported by the CBSG, a Netherlands Genomics Initiative, and the University of Amsterdam.
